# A neuroscientific explanation for the learning effects attributed to Teaching Games for Understanding in physical education and sport coaching practices

**DOI:** 10.3389/fspor.2026.1889606

**Published:** 2026-08-31

**Authors:** Dean A. Dudley

**Affiliations:** 1 School of Human Movement and Nutrition Science University of Queensland, Brisbane, QLD, Australia; 2 Centre of Educational Measurement and Assessment, University of Sydney, Sydney, NSW, Australia

**Keywords:** brain endurance training, cognitive, development, pedagogy, physical literacy

## Abstract

This paper bridges two bodies of literature by proposing that TGfU can be reconceptualised as a form of implicit BET within PE and sports coaching contexts. By exploring potential links between the cognitive and psychomotor outcomes of TGfU and neurocognitive mechanisms proposed within BET, this integration offers a novel explanatory lens for understanding how game-based pedagogy enhances learning and performance. Furthermore, it provides a foundation for future research exploring how deliberate manipulation of cognitive load within TGfU may optimise both learning outcomes and mental resilience in learners.

Contemporary physical education and sports coaching practices have increasingly shifted from technique-driven instruction toward approaches that integrate cognition and action within authentic performance contexts. The Teaching Games for Understanding (TGfU) pedagogical model exemplifies this shift by foregrounding tactical awareness, decision-making, and skill execution within game-based learning environments (1). Rather than isolating motor skills, TGfU situates learning in dynamic, problem-solving contexts where learners must interpret environmental cues, make rapid decisions, and enact appropriate motor responses. This pedagogical structure inherently links cognitive processes with psychomotor execution, suggesting that learning in TGfU is underpinned by integrated cognitive–motor adaptations.

Empirical research has consistently demonstrated that TGfU produces meaningful learning effects across both cognitive, affective, and psychomotor domains. Meta-analytic evidence indicates that TGfU-based interventions yield significant improvements in decision-making and tactical understanding (2), alongside gains in skill execution and overall game performance (3). Importantly, large-scale synthesis work by Dudley and colleagues (4) provides a broader benchmark for interpreting these outcomes. In their systematic review and meta-analysis of 135 physical education interventions involving over 42,000 students, the overall adjusted mean effect size across learning domains was moderate (*d* = 0.40), with psychomotor outcomes demonstrating the strongest effects (*d* = 0.52) and cognitive outcomes comparatively smaller (*d* = 0.17). Notably, the authors identified games-based pedagogies, including TGfU, as among the interventions that consistently exceeded the average effects across both the psychomotor and cognitive learning domains in K–12 students.

These findings seem to suggest that TGfU's effectiveness may lie in its capacity to simultaneously engage learners cognitively and physically, rather than treating these domains as separate. The continuous interaction between perception, decision-making, and motor execution in TGfU environments requires learners to sustain attention, process information under pressure, and coordinate complex motor responses. As such, TGfU can be conceptualised as a pedagogical approach that systematically challenges learners to manage concurrent cognitive and physical demands.

Parallel to developments in pedagogy, interesting advances in sport and exercise psychology have highlighted the critical role of mental fatigue and cognitive effort in shaping physical performance. Mental fatigue (i.e., a psychobiological state resulting from prolonged cognitive exertion) has been shown to impair both cognitive functioning and motor performance (5). To address this issue, Brain Endurance Training (BET) has emerged as a novel intervention designed to enhance resistance to mental fatigue by combining sustained cognitive tasks with physical exercise. BET typically employs dual-task paradigms that require simultaneous engagement of executive functions and physical effort (5).

Early evidence suggests that BET not only improves endurance performance but also reduces the cognitive cost of effort, potentially through adaptations in neural systems associated with attention and self-regulation, including the frontoparietal and salience networks (5). BET researchers imply that the integration of cognitive and physical demands can produce transferable adaptations that enhance both mental resilience and motor output, offering a compelling explanatory framework for performance in complex and dynamic environments.

Despite growing research evidence in both TGfU and BET, these fields of inquiry have been developed in parallel, with no reported conceptual integration to date. However, a clear theoretical convergence is now apparent. The greater than average effect sizes associated with games-based pedagogies (4), particularly within psychomotor and cognitive learning domains, suggest that such approaches may inherently train the capacity to manage cognitive–physical load. TGfU learning environments require sustained attention, rapid information processing, and adaptive decision-making under physical load (2) which are the conditions that closely resemble the dual-task demands central to BET. Consequently, it is plausible that the learning effects observed in TGfU are partially underpinned by mechanisms like those targeted in BET, particularly in terms of increased resistance to mental fatigue and improved efficiency in cognitive–motor coordination.

This paper seeks to bridge these two bodies of literature by proposing that TGfU can be reconceptualised as a form of implicit BET within PE and sports coaching contexts. The term implicit BET is used here to denote a pedagogical environment that may generate cognitive-physical demands analogous to those employed in BET interventions, despite not being intentionally designed as a form of cognitive training. Unlike conventional BET protocols, TGfU does not employ standardised cognitive tasks or seek to directly induce neurocognitive adaptation. Rather, any BET-like effects are hypothesised to emerge as a by-product of authentic game-based learning.

By linking the cognitive and psychomotor outcomes of TGfU to the neurocognitive mechanisms underpinning BET, this integration offers a novel explanatory lens for understanding how game-based pedagogy enhances learning and performance. Furthermore, it provides a foundation for future research exploring how deliberate manipulation of cognitive load within TGfU may optimise both learning outcomes and mental resilience in learners.

## The learning imperative of TGfU

Teaching Games for Understanding emerged as a response to longstanding critiques of traditional approaches to physical education (PE) and sport coaching, which have historically prioritised the decontextualised acquisition of technical skills through repetitive drills and teacher-directed instruction (1). Traditional skill-drill-game approaches to PE and sports coaching are often fragmented learning experiences with techniques taught in isolation from the tactical and perceptual demands of authentic gameplay. This overly reductionist orientation has been criticised for limiting learners’ ability to transfer skills to dynamic performance environments, as it neglects the decision-making processes and contextual awareness required in real game situations (6). Consequently, traditional PE and coaching models have been associated with passive learning, reduced motivation, and limited development of higher-order cognitive skills central to effective participation in sport (7).

By contrast, TGfU represented a paradigmatic shift toward a learner-centred, constructivist pedagogy that explicitly embeds learning within game-based contexts. By structuring learning around modified games classified according to tactical intent, TGfU positions tactical challenges as the starting point for instruction, thereby requiring learners to engage in perception, decision-making, and action simultaneously. According to Bunker and Thorpe (1), this integration reflects the clear learning imperative of the pedagogical/coaching model. Specifically, that the development of understanding the “why” as a precursor to and assessment of skill execution. Over 40 years on, numerous empirical sources support Bunker and Thorpe's (1) claim. There are now meta-analytic findings demonstrating that tactical approaches such as TGfU produce significant improvements in decision-making and other cognitive learning processes when compared to traditional technical PE/sport approaches to instruction (8).

The learning imperative underpinning TGfU is further reinforced by comparative intervention studies that highlight its effectiveness in promoting meaningful and transferable learning outcomes. For example, TGfU-based programmes have been shown to yield greater improvements in decision-making and tactical behaviour than direct instruction models in youth sport contexts (9). While traditional approaches may support initial technical and fundamental movement skill development, TGfU appears to foster deeper learning, including enhanced adaptability, problem-solving capacity, and the ability to apply skills under game conditions (10).

Beyond cognitive and psychomotor outcomes, evidence suggests that TGfU also advances a broader conception of learning that encompasses affective and socialdomains. According to Pan and colleagues (3) and Lodewyk and Bracco (11), TGfU can enhance motivation, enjoyment, and self-efficacy when compared to more controlling, teacher-centred instructional approaches. These researchers argue that engaging learners in meaningful, game-like contexts that emphasise autonomy, collaboration, and problem-solving, TGfU fosters a sense of ownership over learning that is often absent in traditional PE settings.

Collectively, these distinctions position TGfU not merely as an alternative instructional strategy, but as a pedagogical model fundamentally oriented toward learning. Its emphasis on understanding, decision-making, and contextualised skill execution reflects a shift away from the reproduction of technique toward the development of intelligent performers capable of adapting to complex and dynamic environments. As such, TGfU embodies a clear learning imperative that differentiates it from traditional approaches and underpins its growing prominence within PE and sport pedagogy. However, whilst this learning imperative is evident, there have yet to be any neuroscientific process explanations to why these learning effects are so consistently evident. To understand why, we must examine other fields of sport science that have sought to explain the neuroscientific relationships between cognitive and psychomotor learning that occur through physical activities.

## The neuroscience of brain endurance training

Brain Endurance Training (BET) interventions combine cognitively demanding tasks with concurrent or subsequent physical exercise to improve both mental resilience and physical performance capacity. Typically, participants engage in prolonged or repetitive cognitive activities (e.g., response inhibition, working memory, or attention tasks) designed to induce mental fatigue, followed by endurance-based physical exercise such as cycling or running. The underlying premise is that repeated exposure to mental fatigue enhances tolerance to effort-related sensations and improves self-regulation processes, mediated in part by central nervous system adaptations and altered perception of effort (12, 13). Over time, this integrated approach aims to reduce the negative impact of mental fatigue on physical performance by training the brain to better sustain effort under challenging conditions. Empirical evidence suggests that BET induces adaptations distinct from traditional physical training alone, particularly in cognitive control and perception of exertion rather than cardiorespiratory fitness *per se*. For example, improvements are often observed in time-to-exhaustion and endurance performance without significant changes in physiological markers such as VO₂max, indicating a central (brain-mediated) mechanism of adaptation (14, 15). BET interventions are now being increasingly applied in sport and exercise contexts to enhance performance under fatigue, with protocols typically spanning several weeks and incorporating progressively demanding cognitive loads. This approach reflects a growing recognition of the brain's role as a key regulator of endurance performance.

At its core, BET is grounded in a neurocognitive field of inquiry that emphasises the interaction between large-scale brain networks responsible for attention, executive control, and self-regulation. Central to this research is the modulation of three key neural systems being a) the frontoparietal network (FPN), b) the salience network (SN), and c) the default mode network (DMN). The FPN is associated with executive functions such as working memory, attentional control, and task management, while the SN acts in a moderating capacity which detects behaviourally relevant stimuli and the dynamic switching between task-positive and task-negative states. The DMN on the other hand is associated with internally directed cognition and tends to deactivate during externally focused tasks. It is also the region of the brain associated with mind-wandering, day dreaming, and envisioning the future.

BET protocols appear to strengthen the functional connectivity and coordination between these networks, which in turn improve the brain's capacity to sustain goal- directed behaviour under conditions of fatigue (5). BET is hypothesised to enhance the efficiency of this network interplay by suppressing DMN activity and strengthening FPN dominance during cognitively demanding physical performance (5).

Mental fatigue, and by extension the adaptations targeted by BET, have been linked to activity within specific cortical and subcortical regions of the brain. Specifically, the prefrontal cortex (PFC), anterior cingulate cortex (ACC), and basal ganglia. The PFC plays a central role in executive control and the allocation of cognitive resources (16), while the ACC is critically involved in effort monitoring, error detection, and the regulation of perceived exertion (17). During sustained cognitive activity, reduced activation in these regions has been associated with declines in attention and task performance, reflecting the onset of mental fatigue. The basal ganglia, which contribute to motivation and motor control, are also implicated in the regulation of effort and the willingness to persist in demanding tasks (18). BET interventions are thought to induce neuroplastic adaptations within these circuits, improving the efficiency with which cognitive control and motivational processes are deployed during prolonged exertion (5).

A key mechanism through which BET may influence performance is the modulation of perceived effort, a construct closely linked to neural activity in the ACC and insular cortex. Mental fatigue has been shown to increase the subjective perception of effort without necessarily altering physiological capacity, leading to earlier disengagement from physical tasks (19). Neuroimaging evidence indicates that regions such as the dorsal ACC and anterior insula act as central nodes in a “fatigue network,” integrating cognitive, emotional, and interoceptive information to inform decisions about task continuation or cessation (20). BET appears to attenuate this effect by reducing the cognitive cost associated with sustained effort, thereby enabling individuals to maintain performance despite high levels of mental and physical demand (5).

In addition to functional brain networks, neurotransmitter systems may play a critical role in the neurobiology of mental fatigue and the adaptations induced by BET. Prolonged cognitive exertion has been associated with alterations in dopaminergic (21), serotonergic (22), and noradrenergic activity (23), all of which are integral to motivation, mood regulation, and attentional processes. Proponents of BET suggest that this may help stabilise or optimise these neurochemical systems by repeatedly exposing individuals to combined cognitive and physical stressors. In turn, enhancing the brain's capacity to regulate effort and sustain engagement in demanding tasks (24).

Beyond its role in effort regulation, dopamine is also central to the mesolimbic reward pathway involving structures such as the ventral tegmental area (VTA) and nucleus accumbens. This system has been strongly linked to intrinsic motivation, reward anticipation, reinforcement learning, and sustained behavioural engagement. Neurocognitive perspectives suggest that rewarding and meaningful learning experiences may activate these pathways, increasing task engagement and persistence. While BET research has primarily focused on fatigue resistance and effort regulation, the motivational characteristics of TGfU may additionally recruit reward-related neurocognitive processes that support continued participation and learning.

From a systems-level perspective, emerging neuroimaging evidence suggests that mental fatigue is associated with a distributed “fatigue network” encompassing frontal, cingulate, limbic, and parietal regions. This network reflects the integration of cognitive control, emotional processing, and somatosensory feedback, highlighting the multidimensional nature of fatigue (25). BET-induced adaptations are therefore likely to be similarly distributed, involving changes in both regional activation and functional connectivity across this network. By enhancing the efficiency of these interconnected systems, BET may be associated with greater capacity and willingness to exert effort, potentially contributing to enhanced endurance performance in cognitively demanding environments.

When the emerging BET evidence is collectively reviewed, the neuroscience underpinning it suggests that its effectiveness is not solely attributable to improvements in either cognitive or psychomotor capacity in isolation, but rather to the optimisation of neural systems that coordinate these domains. Through repeated exposure to dual-task demands, BET appears to recalibrate the brain's regulation of effort, attention, and motivation, enabling more efficient performance under fatigue. This integrative neural perspective provides a compelling foundation for understanding how cognitive–psychomotor training interventions can enhance human performance, and offers valuable insight for extending such principles into educational and pedagogical contexts.

## Integrating brain endurance training mechanisms with TGfU learning effects

The enhanced learning outcomes associated with TGfU can be meaningfully interpreted through similar neurocognitive mechanisms underpinning BET. At a fundamental level, both TGfU and BET involve the concurrent engagement of cognitive and physical processes, requiring learners to sustain attention, regulate effort, and make contextually appropriate decisions under conditions of uncertainty. BET research indicates that such dual-task demands strengthen functional connectivity across large-scale brain networks that collectively govern attention, executive control, and task engagement (5). Within TGfU contexts, learners are repeatedly exposed to similar cognitive–motor challenges, suggesting that the learning gains observed may be consistent by analogous neural adaptations that enhance the efficiency of these networks during gameplay.

One key explanatory mechanism lies in the optimisation of executive control processes mediated by the FPN. BET protocols specifically target executive functions such as working memory, inhibitory control, and sustained attention, which are essential for maintaining performance under cognitive strain (5). TGfU similarly places high demands on these functions, as learners must continually process evolving game information, inhibit inappropriate responses, and select optimal actions in real time. Repeated engagement in such cognitively demanding environments may contribute to adaptations that are consistent with increased neural efficiency within the FPN, resulting in improved decision-making and tactical awareness which are learning outcomes prioritised in the TGfU pedagogical model (1). It is therefore plausible that the cognitive gains associated with TGfU (4) can be interpreted as emergent properties of enhanced executive functioning shaped by sustained cognitive–physical coupling.

A second plausible mechanism of understanding this phenomenon relates to the role. of the SN in allocating attentional resources and facilitating adaptive responses to changing environmental demands. The SN is responsible for detecting behaviourally relevant stimuli and coordinating the switch between internal (DMN) and external (FPN) modes of processing (5). In TGfU settings, where learners must constantly respond to dynamic and unpredictable game situations, efficient salience detection is critical. BET-induced improvements in SN functioning may therefore translate to improved perceptual acuity and faster, more accurate decision-making in TGfU contexts. This aligns with evidence that TGfU enhances players' ability to perceive and act upon tactical affordances within gameplay (26).

The regulation of perceived effort provides a further neurobiological link between BET and TGfU learning outcomes. Mental fatigue has been shown to increase perceived exertion through activity in the ACC and insular cortex, leading to reduced persistence and impaired performance (27). BET appears to attenuate this relationship by reducing the cognitive cost of effort and enhancing tolerance to mental fatigue (5). When TGfU is the pedagogical approach to teaching and coaching, learners are frequently required to sustain cognitive engagement over extended periods of gameplay. The ability to regulate perceived effort and resist mental fatigue may therefore enable learners to maintain higher levels of cognitive and motor performance throughout practice, facilitating

deeper learning and more robust skill acquisition.

Neurochemical adaptations further support this interpretation in the same way BET seems to contribute to the stabilisation of dopaminergic function through repeated exposure to cognitively demanding tasks, thereby enhancing motivation and persistence (24). In TGfU-based activities, where learning is embedded within meaningful and engaging game contexts, such neurochemical adaptations may be a result of the learning and movement behaviour inculcalted in TGfU pedagogical design that in turn facilitates sustained engagement and intrinsic motivation, reinforcing learning processes across cognitive and psychomotor domains simultaneously.

An additional explanatory mechanism may relate to the mesolimbic reward system. TGfU learning environments are intentionally designed around meaningful game play, challenge, autonomy, and social interaction. These features have been associated with enhanced enjoyment, motivation, and self-efficacy in TGfU interventions (3, 11). From a neurocognitive perspective, it is plausible that such experiences engage reward-related pathways involving dopaminergic projections between the ventral tegmental area and nucleus accumbens. Although direct evidence in TGfU contexts is currently lacking, the activation of reward-related systems may provide an additional explanation for the sustained engagement and persistence commonly reported in game-based learning environments.

Finally, both BET and TGfU can be understood from a systems perspective as interventions that recalibrate the broader “fatigue network” of the brain, encompassing frontal, cingulate, limbic, and parietal regions involved in attention, emotion, and somatosensory processing (25). If similar adaptations occur within TGfU contexts, learners become better equipped to manage the cognitive and physical demands of performance environments. In TGfU, this may manifest as enhanced adaptability, more consistent decision-making, and improved execution under pressure. All of which are key indicators of high-quality learning and performance in both PE and coaching contexts.

## Conclusions and implications

This paper argues that it is plausible that the neural mechanisms associated with BET provide a robust explanatory framework for understanding the enhanced learning effects observed in TGfU across the globe. Through the optimisation of large-scale brain networks, improved regulation of effort and fatigue, and neurochemical adaptations supporting motivation and persistence, BET offers insight into how integrated cognitive–physical training environments may drive superior learning outcomes. TGfU, when viewed through this neurobiological lens, can be conceptualised as an ecologically valid, pedagogical instantiation of brain endurance training. It is a teaching/coaching strategy that naturally cultivates the neural capacities required for effective learning and performance in complex and dynamic contexts.

The conceptual alignment between BET and TGfU carries significant implications for pedagogical practice in PE and sport coaching. If TGfU learning environments share features that resemble the dual-task demands characteristic of BET, then their effectiveness may be interpreted not only in pedagogical terms but also through a neurocognitive lens. BET research demonstrates that the coupling of cognitive and physical load enhances resistance to mental fatigue and improves the efficiency of neural systems underpinning attention, executive control, and effort regulation (5). Such findings suggest that TGfUs emphasis on authentic, decision-rich gameplay may align with neurophysiological mechanisims believed to support learning.

From a curriculum design perspective, this insight reinforces the value of maintaining high levels of cognitive engagement within PE and coaching contexts. Traditional approaches that isolate technique and reduce cognitive demands may inadvertently limit the development of neural systems associated with decision-making and effort regulation. By contrast, TGfU's use of modified games, tactical questioning, and problem-solving tasks ensures that learners are consistently required to engage

executive functions, including sustained attention and inhibitory control (3). When interpreted through a BET lens, these strategies can be seen as systematically training the brain's capacity to allocate cognitive resources under physical load and thereby enhancing both learning and performance outcomes.

For practitioners, this integration suggests a need to deliberately manipulate cognitive load alongside physical intensity in lesson and session design. The concept of cognitive load as a critical pedagogical variable in PE environments has been previously raised by Dudley and colleagues (28), who argue that learning in physically active contexts is fundamentally constrained by the limited capacity and duration of working memory. From a cognitive load theory (CLT) perspective, effective learning depends on the successful transfer of information from working memory into long- term memory through schema construction (29); however, when ***instructional*** demands exceed working memory capacity, learning may be compromised (27). Importantly however, the cognitive demands imposed by TGfU are unlikely to be experienced uniformly across learners. Cognitive load theory suggests that working memory limitations interact with prior knowledge and expertise, meaning that game-based challenges that are appropriately demanding for one learner may be overwhelming or insufficiently challenging for another. Individual differences in cognitive capacity, developmental stage, and affective characteristics may therefore moderate the effectiveness of cognitively demanding pedagogical environments.

Within TGfU environments, where learners are simultaneously required to perceive dynamic game situations, process tactical information, and execute motor responses, the occurrence of cognitive overload is particularly pronounced. Dudley and colleagues (28) emphasise that the physical literacy impetus of PE requires learners to draw on multiple domains of learning (i.e., cognitive, physical, affective, and social) thereby increasing germane cognitive load and making hence making instructional design and delivery a critical determinant of learning success. This suggests that the effectiveness of TGfU may not simply be a function of its game-based design, but rather its capacity, if well implemented, to manage cognitive load in ways that optimise learning.

In practical terms, this necessitates a more deliberate and nuanced approach to task design, where teachers and coaches actively regulate both intrinsic and extraneous cognitive load. For example, intrinsic cognitive load can be managed by modifying the complexity of game tasks (e.g., reducing player numbers, simplifying rules, or segmenting tactical problems), allowing learners to progressively build schemas without overwhelming working memory. At the same time, the extraneous load arising from poorly structured instruction or unclear task demands, must be minimised (27).

Furthermore, Dudley and colleagues (28) highlight the importance of scaffolding and sequencing learning experiences, particularly via Load Reduction Instruction (LRI). When applied to TGfU, this suggests that while game-based learning promotes cognitive engagement, it must be carefully scaffolded, especially for novice learners, to avoid excessive cognitive demands that may impair schema formation. Conversely, as learners develop expertise and automaticity, reducing instructional guidance and increasing autonomy becomes critical to avoid the “expertise reversal effect,” whereby overly explicit instruction can hinder learning for more advanced performers.

These insights reinforce the idea that cognitive load is not merely a by-product of TGfU learning environments, but a central pedagogical lever that can be strategically manipulated to enhance learning outcomes. When aligned with the dual-task demands identified in BET, the deliberate calibration of cognitive load within TGfU has the potential to optimise both neural adaptation and educational outcomes, supporting the development of learners who are not only physically competent, but also cognitively resilient and capable of sustained performance in variety of volatile, uncertain, complex, and ambiguous environments.

In addition, the intersection of BET and TGfU thinking highlights the importance of recognising mental fatigue as a pedagogical variable. PE teachers and coaches may need to consider how task sequencing, lesson structure, and recovery periods influence learners' cognitive states. Within TGfU, this could involve balancing high- cognitive-load activities with opportunities for reflection and recovery, ensuring that learners remain within an optimal zone of cognitive challenge that promotes adaptation without excessive fatigue.

If TGfU is conceptualised as an implicit form of BET, it should also support a shift toward more integrated models of learning in PE and sport coaching. Rather than viewing physical, cognitive, social, and affective domains as separate, this perspective emphasises their interdependence and the role of pedagogical design in shaping their interaction. This aligns with emerging evidence from meta-analytic work demonstrating that games-based approaches produce above-average effects across multiple learning domains, particularly in psychomotor and cognitive outcomes (4) and the learning imperative of physical literacy in the construction of successful educational and sporting contexts (30–32). By situating these findings within a neuroscientific framework, educators and coaches gain a more comprehensive understanding of why such approaches are effective and how they can be optimised.

A limitation of the present conceptualisation is that it necessarily treats learners as a relatively homogeneous population. However, both cognitive load theory and contemporary neuroscience suggest that responses to cognitively demanding physical activities are likely moderated by individual learner characteristics. Factors such as age, prior expertise, working memory capacity, motivation, and affective dispositions may influence how learners experience and adapt to the cognitive-physical demands inherent in TGfU environments. Consequently, any potential BET-like effects associated with TGfU should not be assumed to occur uniformly across all learners.

## Future research

To further investigate the relationship between BET and TGfU, future research should adopt interdisciplinary designs that integrate pedagogical, performance, and neuroscientific measures. This might include quasi-experimental or randomised controlled trials comparing TGfU-based interventions with traditional technical approaches, while systematically manipulating cognitive load. Participants could be assigned to conditions that vary in both pedagogical approach (TGfU vs. technical instruction) and cognitive demand (low vs. high), creating a factorial design capable of isolating the effects of cognitive–physical integration.

Outcome measures should extend beyond traditional assessments of skill execution and game performance to include indices of cognitive function (e.g., executive function tests), perceived effort (e.g., Rating of Perceived Exertion scales adapted for mental effort), and markers of mental fatigue. Where feasible, neurophysiological measures such as functional near-infrared spectroscopy (fNIRS) or electroencephalography (EEG) could be employed to examine changes in cortical activation and functional connectivity, particularly within networks implicated in BET (e.g., FPN, SN, DMN). Such measures would allow researchers to directly test whether TGfU interventions produce neural adaptations consistent with those observed in BET studies.

Longitudinal designs would also be particularly valuable, as they would enable the examination of how sustained exposure to TGfU influences the development of cognitive resilience and fatigue resistance over time. Additionally, mediation analyses could be used to explore whether changes in cognitive or neural variables explain improvements in learning and performance outcomes. This would provide critical insight into the causal mechanisms linking pedagogical practice and learning effects.

Finally, future studies should consider ecological validity by situating interventions within authentic PE and coaching settings, while maintaining sufficient experimental control to isolate key variables. Mixed-methods approaches incorporating qualitative data (e.g., student or athlete perceptions of cognitive effort and engagement) may also provide some valuable insights into the experiential dimensions of cognitive–physical learning.

The integration of BET and TGfU offers a compelling framework for rethinking pedagogy in PE and sport coaching. By recognising that effective learning environments are those that challenge both the body and the brain, educators and coaches can design practices that promote not only skill acquisition but also cognitive resilience and adaptability. Future research that systematically investigates this intersection has the potential to advance both theoretical understanding and practical application, ultimately contributing to more effective and holistic approaches to teaching and coaching.
